# Quantitative Proteomics of Potato Leaves Infected with *Phytophthora infestans* Provides Insights into Coordinated and Altered Protein Expression during Early and Late Disease Stages

**DOI:** 10.3390/ijms20010136

**Published:** 2019-01-01

**Authors:** Chunfang Xiao, Jianhua Gao, Yuanxue Zhang, Zhen Wang, Denghong Zhang, Qiaoling Chen, Xingzhi Ye, Yi Xu, Guocai Yang, Lei Yan, Qun Cheng, Jiaji Chen, Yanfen Shen

**Affiliations:** 1 Southern Potato Research Center of China, Academy of Agricultural Sciences, Enshi 445000, Hubei, China; 15971827976@163.com (C.X.); wend315@126.com (J.G.); 13636289689@163.com (Y.Z.); wangzhen20093332@163.com (Z.W.); zhangdenghong2@126.com (D.Z.); 18627798503@163.com (Q.C.); yxz161718@163.com (X.Y.); enshizizi@126.com (Y.X.); ygcdxs@126.com (G.Y.); 18727670407@163.com (L.Y.); enshicq@126.com (Q.C.); chenjia-ji@163.com (J.C.); 2 Enshi Tujia and Miao Autonomous Prefecture Academy of Agricultural Sciences, Enshi 445000, Hubei, China

**Keywords:** late blight disease, potato proteomics, *Phytophthora infestans*, Sarpo Mira, early and late disease stages

## Abstract

In order to get a better understanding of protein association during *Solanum tuberosum* (cv. Sarpo Mira)–*Phytophthora infestans* incompatible interaction, we investigated the proteome dynamics of cv. Sarpo Mira, after foliar application of zoospore suspension from *P. infestans* isolate, at three key time-points: zero hours post inoculation (hpi) (Control), 48 hpi (EI), and 120 hpi (LI); divided into early and late disease stages by the tandem mass tagging (TMT) method. A total of 1229 differentially-expressed proteins (DEPs) were identified in cv. Sarpo Mira in a pairwise comparison of the two disease stages, including commonly shared DEPs, specific DEPs in early and late disease stages, respectively. Over 80% of the changes in protein abundance were up-regulated in the early stages of infection, whereas more DEPs (61%) were down-regulated in the later disease stage. Expression patterns, functional category, and enrichment tests highlighted significant coordination and enrichment of cell wall-associated defense response proteins during the early stage of infection. The late stage was characterized by a cellular protein modification process, membrane protein complex formation, and cell death induction. These results, together with phenotypic observations, provide further insight into the molecular mechanism of *P. infestans* resistance in potatos.

## 1. Introduction

Potato late blight disease caused by *Phytophthora infestans* is one of the most critical crop diseases in the world. Late blight was responsible for the European potato famine in the 19th century [1]. It poses a severe threat to potato production worldwide, with estimated annual economic losses of over six billion dollars, mainly due to yield loss and the high cost of fungicide [2]. Management of the late blight disease pathogen is challenged by global warming, environmental regulations against the use of chemical fungicides, and *P. infestans* remarkable pathogenicity [3].

*P. infestans* shows an initial asymptomatic biotrophic phase of infection followed by a necrotrophic phase. During the biotrophic stage, *P. infestans* forms appressoria, primary and secondary hyphae, and specialized structures called haustoria, through which effectors are delivered into the host apoplast or adjacent cells [4]. Plants have evolved an array of innate immune systems to detect and respond to a wide range of these *P. infestans* effectors. For example, PTI (Pathogen-associated molecular pattern-triggered immunity), which uses transmembrane pattern recognition receptors (PRRs) that respond to evolving microbial- or pathogen-associated molecular patterns (MAMPS or PAMPs). PRRs include a class of leucine-rich repeat (LRR)-receptor kinases (RK), for example CEBiP [5] and OsCERK1 [6,7] in rice; AtCERK1 [8,9,10,11], LYM2 [12,13,14], RBGP1 [15], and RLP30 [15] in Arabidopsis; and EIX2 [16], Ve1 [17,18], and Cf-9 [15]. However, knowledge about PRRs in the potato host is scarce. PTI has the potential to fend off various microbes, pathogenic or not, due to the conserved nature of PAMPs (e.g., fungal chitin) across species, genera, family, or class. Thus, PRRs can provide resistance to most non-adapted pathogens, as well as contribute to basal immunity during infection or disease process. In response to these PTI defense systems, pathogens that could breach PTI successfully deploy a huge number of effectors to render pathogen virulence. Such effectors change the normal function of PTI, resulting in effector-triggered susceptibility (ETS) [19]. A large number of extracellular and cytoplasmic effectors in the *P. infestans* genome have been identified and increasing evidence for their role in establishing ETS exists [20,21].

However, to combat pathogens with established ETS, host plants have evolved a race-specific immunity, a well-described host resistance mechanism that is governed by dominant R-genes. Many R-genes have been cloned, and most of them encode proteins with N-terminal nucleotide-binding sites (NBSs) and C-terminal leucine-rich repeats (LRRs). R-genes encode proteins that recognize pathogen effectors to establish effector-triggered immunity (ETI). This recognition triggers a cascade of defense responses, mediated by a complex-signaling network, in which plant hormones, like salicylic acid (SA) and jasmonic acid (JA), play a significant role and the resistance is manifested as a localized hypersensitive cell death response (HR) at the site of infection.

Recently, next-generation sequencing (NGS) technologies are transforming biology research [22,23]. Genome sequences of potato and *P. infestans* have been published [2,24], making sequencing-based “omics” studies more accessible to potato late blight researchers. Proteomics has become a viable alternative for molecular analysis, providing information and tools for a better understanding of the plant-pathogen relationship. Recently, proteomics has dramatically evolved in the pursuit of large-scale functional assignment of candidate proteins and, by using this approach, several proteins expressed during potato–*P. infestans* interaction have been identified [25,26,27]. Two-dimensional electrophoresis (2-DE) based proteomics [25], gel-based protein shotgun mass spectrometer [28], and, most recently, label-free proteomics analysis [26,27], have shed light on our understanding of compatible and incompatible interactions between *P. infestans* and potato.

The recently developed isobaric label proteomics, such as isobaric tags for relative and absolute quantification (ITRAQ) [29] and tandem mass tags (TMT) [30], are chemically conjugated to the primary amines of peptides after tryptic digestion and are compatible with samples from multiple sources [29]. Therefore, in this study, we used the TMT method to measure and compare the changes in protein abundance of potato cv. Sarpo Mira after foliar application of zoospore suspension of *P. infestans* at three key timepoints, covering potato–*P. infestans* oomycete early (EI) and late (LI) stage of interaction. A total of 1229 differentially-expressed proteins (DEP) were identified, 75 DEPs at the early stage and 723 DEPs at the late stage of the disease process. The proteins identified at the early and late stage could play an essential role in early pathogen recognition, signal transduction, disease resistance processes against *P. infestans*, and possibly disease pathogenesis. This study will contribute to a better understanding of the molecular mechanism of *P. infestans* interaction with potato.

## 2. Results and Discussion

### 2.1. Subsection Phenotypic Differences between the Three Stages of Disease Conditions

**Figure 1:**
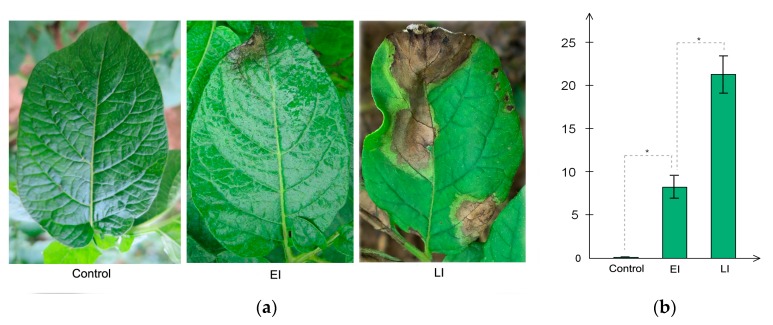
Phenotypic observation of the three time-points. Control corresponds to time-point 0 Hours post inoculation, EI corresponds to 48 hpi, and LI corresponds to 120 hpi. (**a**) Sizes of lesions induced by the *P. infestans* zoospores at different exposure times. The diameter of each lesion was measured at 2 and 5 days after inoculation (**b**). Three replicates were used for each treatment in these tests. Bars represent the standard deviation of three replicates. Statistical significance was analyzed using Student’s *t*-test. The asterisk indicates the significant difference (* *p* < 0.05).

A time series assessment of Sarpo Mira leaf phenotype challenged with *P. infestans* is shown in Figure 1a,b. Three replicates were used for each treatment in these tests. After spraying the whole potato plant with *P. infestans* zoospores, there was no observable microscopic hypersensitive reaction (HR) lesions at 0 Hours post inoculation hpi (Control); however, by 48 hpi (EI), HR lesions had appeared, which resulted in a localized necrosis that resembled a *P. infestans*-induced hypersensitive response, and at 120 hpi (LI) the leaves had developed much larger H -induced necrosis, consistent with a typical R gene-mediated HR lesions expansion, as previously reported in Sarpo Mira [31]. There was a significant difference in the lesion size of LI relative to EI, as seen in (Figure 1b), and consistent with previous studies [32,33]. Based on these results, we designated the interval between time-points Control and EI as early disease stage and timepoint EI to LI as late disease stage. 

### 2.2. Overview of Protein Expression in Potato Leaves Challenged with P. infestans Oomycete

**Figure 2:**
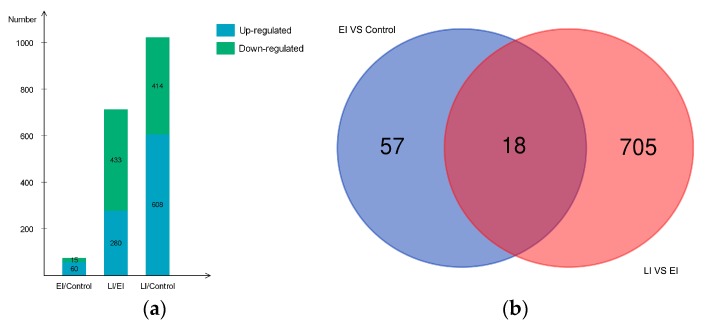
Differentially expressed proteins between the early and late stage (**a**). Bar chart showing the number of up- and down-regulated proteins in each of the pairwise comparisons: EI/Control, LI/EI, and LI/Control. green color indicates down-regulated proteins and blue color indicates up-regulated proteins. Overlaps among differentially expressed proteins (DEPs) between the early and late stage (**b**). Venn diagrams depict overlap of DEPs from each pairwise comparison between the timepoints, EI/Control (early) and LI/EI (late).

Potato cv. Sarpo Mira was previously reported to have incompatible interaction with *P. infestans* [29]; however, systematic analysis of protein association during Sarpo Mira–*P. infestans* interaction, and their resultant changes in abundance leading to incompatibility, are incompletely understood. Therefore, to shed more light on the changes in protein abundance during the early and late stages of late blight disease, we performed a comparative proteome survey by TMT method [29] at three key time-points (Control, EI, and LI time-points. See method section) on leaves of potato clone Sarpo Mira inoculated with *P. infestans* oomycete. Three biological replicates were collected at the same time. With these measurements, a total of 15,813 high-quality peptides (in at least two replicates per time-point) corresponding to 4643 proteins were identified in the time series analysis (Table S1). Of the 4643 proteins, 1229 (at least one unique peptides) were found to be differentially expressed proteins (DEP) in a pairwise comparison between the time-points. Among the 1229 DEPs, 952 had functional annotations (Table S2). In all, a total of 1082 DEP could be classified into 70 different significantly-enriched protein domains and features by InterProScan analysis, 21 PFAM protein domains, and 56 significantly-enriched Kyoto Encyclopedia of Genes and Genomes pathways (Table S2) [34]. The total number of DEPs observed in each pairwise comparison is shown in Figure 2a and Tables S3–S5. For example, 1022 DEPs were observed in the group pair of H/L, covering the whole-time course. The pair of EI/Control had 75 proteins representing DEPs in the early stage of pathogen invasion, and LI/EI had 723 DEPs active during the late stage of the disease process.

Large scale comparative quantitative proteomic studies produce numerous lists of proteins containing biological identifiers, and often it is useful to highlight the overlapping sets between groups of biological data, enabling quick and easy observation of the similarities and differences between the data sets. In this study, dataset overlap between the early and late stage revealed about 2.3% (18/780) of the DEPs were commonly-shared induced proteins throughout the time course (Figure 2b), suggesting that these proteins could be necessary for the sustained HR phenotype observed in the later stages of the disease condition. More strikingly, 90.4% (705/780) of DEPs were specific to LI/EI, while 7.3% (57/780) was unique to EI/Control (Figure 2b).

### 2.3. Gene Ontology Classification of Differentially Expressed Proteins

**Figure 3:**
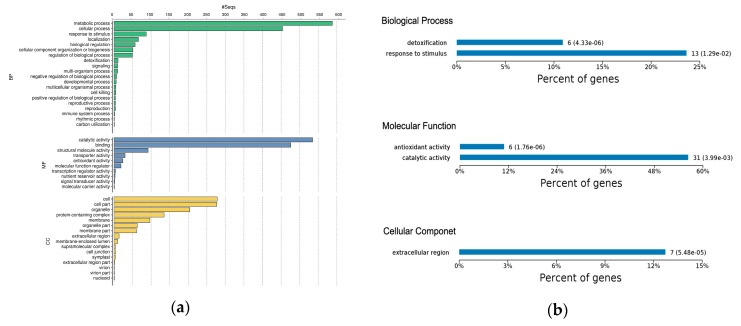

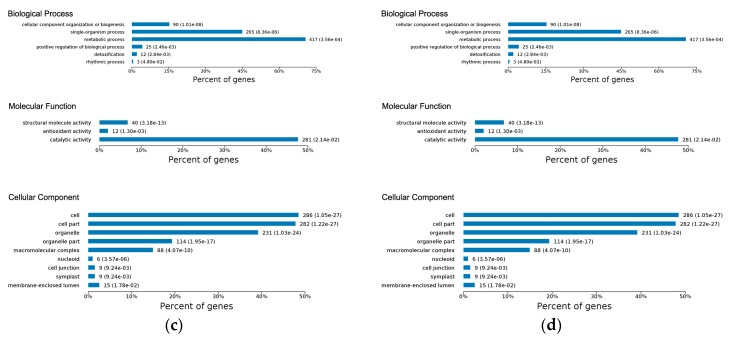
(**a**) Gene Ontology functional classification of all DEPs; bar chart shows the distribution of differentially-expressed proteins among the GO biological process (BP), molecular function (MF), and cellular component (CC). (**b**) GO-based functional enrichment analysis of DEPs at early disease stage. (**c**) GO-based functional enrichment analysis of DEPs at late disease stage. (**d**) Kyoto Encyclopedia of Genes and Genomes pathway enrichment of DEPs at the early and late disease stages.

Gene ontology classification and KEGG (Kyoto Encyclopedia of Genes and Genomes) analysis was used to reveal the implication of all DEPs identified in this study (Figure 3a). Gene Ontology analysis showed that much more biological process categories were highly abundant in the DEPs. The protein classes “Metabolic processes” (41.3%), “Cellular processes” (32%), and “Response to stimulus” (6.1%) were the most abundant categories (Figure 3a). The label “Metabolic process” covers several sub-biological process categories; notable among them were primary and secondary metabolism, such as carbohydrate, lipid, protein, amino acid, and nucloeobase-containing compound metabolism. Also, within this category are systemic-acquired resistance and defense response to fungus. The proteins involved in this group were mostly up-regulated in the early disease stage. The “Cellular processes” includes protein folding, microtubule-based process, signal transduction, cell death, protein secretion cellular homeostasis, and oxidant detoxification. “Response to stimulus“ covered response to stress, response to biotic and abiotic stimulus, response to endogenous stimulus, and response to chemical. Several of these proteins encoded peroxidase, pectinesterase, and endochitinase-like compounds, and were also up-regulated in both disease stages (Figure 3a and Table S6).

Molecular function classification highlighted over-representation of “Catalytic Activity” (45%) and “Binding” (40%) as the major function of the DEPs. The “Catalytic Activity” heading included oxidoreductase activity, transferase activity, hydrolase activity, lyase activity, isomerase activity, and ligase activity. The “Binding” category encompassed protein binding, protein-containing complex binding, ion binding, drug and cofactor binding, iron-sulfur cluster binding, heterocyclic compound binding, glutathione binding, and carbohydrate derivative binding (Figure 3a and Table S6). The majority of the proteins in this category were uncharacterized. Other molecular function categories worthy of mention include antioxidant activity, enzyme regulator activity, and nuclear import signal receptor activity. About half of the DEPs were in the cellular component, of which the majority were in the “Cell” (24.10%), which included the extracellular region and apoplast. “Cell part” (23.96%), covering intracellular plasma membrane and chloroplast envelope; “Organelle” (17.65%), covering organelle membrane, intracellular organelle, and organelle lumen; “Protein complex” (11.68%), including THO complex, U1 snRNP, nucleosome, transcription factor complex, chloroplastic endopeptidase Clp complex, and eukaryotic translation elongation factor 1 complex; “Membrane” (8.39%) and “Membrane part” (5.28%), which encompasses membrane protein complex and inner mitochondrial membrane protein complex (Figure 3a and Table S6).

A functional enrichment test was used to identify over-represented proteins that may have an association with early and late disease phenotypes, by interrogating the data for the GO enrichment of protein sets. The KEGG database was used to determine significantly enriched pathways in the early or late disease stage (Table S6).

In the early disease stage, the GO enrichment test revealed response to stimulus and detoxification as the most significantly-enriched biological processes (Figure 3b). The significantly-enriched molecular functions were antioxidant activity and catalytic activity, which included peroxidase activity (Figure 3b), while several of these enriched proteins were located within the extracellular region of the cellular component (Table S6). Considering the late disease stage, we observed that metabolic process and single-organism process were highly significantly-enriched biological processes (Figure 3c). Other enriched biological processes included detoxification, cellular component and biogenesis, and positive regulation of biological processes (Table S6). Enriched molecular functions included structural molecular activity, antioxidant activity, and catalytic activity (Figure 3c). In the cellular component, we noticed overrepresentation of proteins in the cell, cell part, organelle, and organelle part (Figure 3c).

Pathway coverage analysis using the KEGG database found that the phenylpropanoid biosynthetic pathway was the most significantly-enriched in the early disease stage (Figure 3d); most of these proteins encoded peroxidase/peroxidase-like and hydroxycinnamyl proteins. Other enriched pathways included fatty acid metabolism and biosynthesis of unsaturated fatty acid (Figure 3d). As reported by others, the phenylpropanoid pathway is essential to plants because of its role in the production of the hydroxycinnamyl alcohols, which serve as the building blocks of lignin, and confers structural support, vascular integrity, and pathogen resistance to plants [35]. Additionally, high induction of several genes mapped to the phenylpropanoid pathway has been reported following *P. infestans* invasion [36]. Meanwhile, biosynthesis of secondary metabolism, ribosome, glutathione metabolism, biosynthesis of amino acid, porphyrin and chlorophyll metabolism, ribosome biogenesis, valine, leucine and isoleucine degradation, synthesis and degradation of ketone bodies, and fatty acid metabolic pathways were identified as the most significantly-enriched pathways in the late disease stage (Figure 3d). Together these results suggested that the identified proteins represent a functionally-active subset of the entire proteome associated with the potato response to *P. infestans* oomycete infection.

### 2.4. Differential Expression Pattern of Proteins Involved at the Early and Late Disease Stages

To better understand potato–*P. infestans* interaction, it is important to distinguish the potato’s specific response to the invading pathogen and protein signatures at various stages of the disease process, which can help shed light on the pathogenic life style, whether in biotrophic relationships (in which the pathogen feeds from living host cells) to necrotrophic associations (in which the microbe feeds on nutrients released from killed cells [37]). Therefore, we examined the expression pattern of proteins involved at the early and late disease stages. 

#### 2.4.1. Early Disease Stage Response Proteins

**Figure 4:**
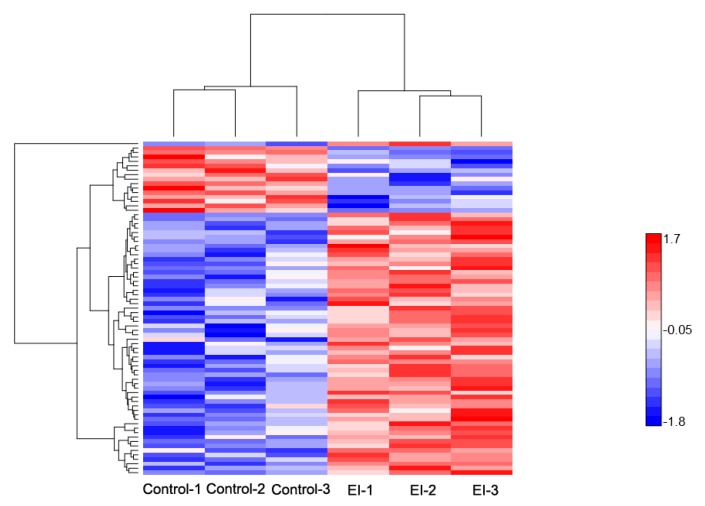
Hierarchical clustering of differentially-expressed proteins at the early disease stage. Heat map showing the changes in protein expression: Proteins with high expression levels (red); proteins with low expression level (blue).

As stated earlier, 75 proteins showed a significant difference in protein abundance at the early stage of the disease process (EI/Control-compared proteins, Table S3), of which 60 DEPs were up-regulated, and 15 DEPs were down-regulated (Figure 2a); their expression profile is shown in (Figure 4). GO enrichment test results (False Discovery Rate < 0.05, *p* < 0.01) revealed these proteins were mostly related to detoxification and response to a stimulus, which covers stress response, defense response, oxidative burst, and cellular catabolic process (Figure 3b). Further analysis uncovered enriched proteins were most active in the apoplast, cell wall, cell periphery, and external encapsulating structure during pathogen invasion (Table S6). To elucidate cv. Sarpo Mira response to *P. infestans* at the initial stages of infection, we further examined the expression pattern and the role of DEPs in EI/Control comparison (Table S3). We found a general trend among the DEPs in EI/Control comparison, here most of the proteins significantly increased in abundance from timepoint L to M (Figure 4). Also, we noticed specific enrichment of positively regulated (from time-point Control to EI) functional categories related to defense and oxidative stress response. The most prominent early response proteins present in this group were cell wall degrading enzymes, for example, wall-associated kinase, annexin, osmotins, osmotin-like proteins, serine protease, and proteinase inhibitors, pectinesterase and putative endochitinase; the later are cell-wall degrading enzymes (CWDEs). We also observed five highly up-regulated peroxidase proteins involved in the reactive oxygen species (ROS) metabolic process. The rapid production of reactive oxygen species (ROS) upon pathogen attack has been associated to the defense mechanism for microbial killing and early initiation of host defense responses in plants [38]. In the present study, the up-regulation of these five peroxidase proteins suggests the induction of oxidative burst, which is typically associated with PTI and HR-specific induced cell death, which is consistent with the phenotypic observation (Figure 1a) and previous studies [33].

We also noticed other proteins, including the specific up-regulation of glucan endo-1,3-beta-glucosidase and serine carboxypeptidase-like-33. In this regard, it is well known that extracellular enzymes of plant pathogenic fungi (e.g., glucanases) may have a diversity of roles in host invasion and pathogenesis, either as an inducer or suppressor [39]. Indeed, there are reports that cell walls of oomycetes consist mainly of (1/3)-b-d-glucans, (1/6)-b-d-glucans, and cellulose, which might be required for normal appressorium formation and successful infection of the potato [39]. However, the specific up-regulation of glucan endo-1,3-beta-glucosidase suggests that it either played a significant role in facilitating *P. infestans* penetration of the host cell wall, or the destruction of papillae, blocking the invading pathogen by releasing glucans from the host wall polymers or by hydrolyzing biologically-active glucans, which could act as elicitor. In contrast it may digest wall components of the invading fungal pathogen [40]. Serine carboxypeptidase belonged to a large family of hydrolyzing enzymes, which are believed to play roles in processing and degradation of proteins/peptides, and studies have shown that this protein family are typically up-regulated during pathogen invasion [39]. In the current study, it may be part of the fungal mechanisms of efficient protein digestion during invasion or a past host cell proteolytic machinery against *P. infestans* [41,42]. Additionally, we identified another set of DEPs that showed significant up-regulation at the early stage, whose domains possess a binding function possibly involved in the production of antimicrobial compounds. They include the pathogenesis-related protein PR-10 family and the NtPRp27-like protein, which suggests a distinct counter-defense mechanism because most PR proteins are reported to exhibit direct antimicrobial activities and may play a role in both constitutive and induced basal defense responses [43].

Contrastingly, few DEPs were repressed at this stage; notable among them are significantly down-regulated proteins such as dehydrin, pyruvate dehydrogenase, light-inducible tissue-specific protein, and three uncharacterized proteins, M0ZKB0, M1AM40, and M1BUI4 (Table S3). These proteins have functions related to amino acid metabolism and transport, sterol biosynthesis, and abscisic acid signaling. To put these results into perspective, the increased abundance of the majority (60/75) of DEPs in EI/Control indicated an early activation of cell wall-associated defense proteins involved in signal transduction, deployment of basal resistance, and initiation of R gene-mediated resistance processes, which reflects a coordinated activation and repression of specific cell wall-associated proteins to correlate with the precise cellular defense requirement to restrict *P. infestans* invasion.

#### 2.4.2. Late Disease Stage Response Proteins

**Figure 5:**
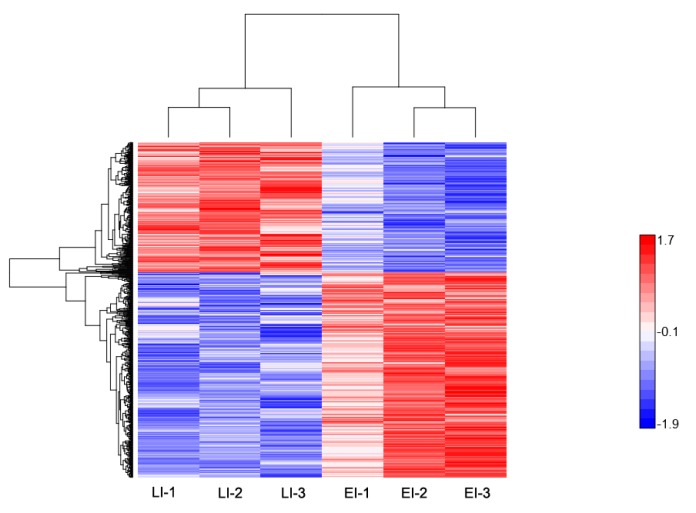
Hierarchical clustering of differentially-expressed proteins at the late disease stage. Heat map showing the changes in protein expression: proteins with high expression levels (red); proteins with low expression level (blue).

The profile of the 723 DEPs identified during the late stage is reported in Figure 5. Within this group, a total of 280 DEPs were up-regulated and 443 were significantly down-regulated, as shown in Figure 2a and Table S4. Functional enrichment showed that the majority of the proteins were involved in cellular component organization, metabolic process, and single-organism process, which encompasses cellular protein modification process and membrane protein complex formation (Figure 3c and Table S6). We noticed a consecutive up-regulation of several proteins with the binding function, including the resistance (R) gene product containing CC-NB-ARC and LRR domains, as well as chitin-binding domains. Typically, R proteins are conserved across the plant kingdom, and have been shown to mediate the resistances of race-specific diseases in plants by recognizing effectors and initiating effector triggered immunity (ETI) [20,21,38]. Several other proteins that were significantly up-regulated were identified within this group, which included proteins possessing acid phosphatase-class B domain, osmotin/thaumatin-like domain, endochitinase activity, and kunitz proteinase inhibitor. The latter is a part of potato proteolytic enzyme inhibitors, which may play an important role in the natural defense mechanisms of the potato plant against phytopathogen attack [27,44] In addition to the above were hyoscyamine-beta-hydroxylase (H6H), Puroindoline-A (PIN-A), Puroindoline-I (PIN-I), lipoxygenase, and hydroxy-methylglutaryl coenzyme A reductase (HMGR). H6H is an enzyme belonging to the family of oxidoreductases, and the last rate-limiting enzyme directly catalyzing the formation of scopolamine in the tropane alkaloids (TAs) biosynthesis pathway [45]. Earlier studies have shown that H6H was concurrently significantly up-regulated, among other R gene proteins, following *P. infestans* treatment in resistant potato cultivars relative to susceptible genotypes [46]. PIN-A and PIN-I are transmembrane proteins involved in auxin efflux [47]; their specific role in potato–*P. infestans* interaction is not clear. Similarly, HMGR is known to be strongly induced by fungal elicitors in rice [45]. In the present study, and generally, it is likely that these proteins together might play a role in the production of highly complex toxic anti-microbial or anti-fungal compounds as defense against invading pathogens [46].

To survive in a peroxidative environment, microbes produce natural antioxidants within host cells, or modulate host cells to produce protectants, including vitamin C, glutathione (GSH) carotenoids, reductases, peroxidases, and several others [48,49]. Here, we identified proteins that are possibly associated with these processes mentioned above and may be implicated in the expansion of late-stage disease processes. These included: peroxidase family proteins and threonine dehydratase, an enzyme involved in isoleucine biosynthesis by catalyzing the deamination of threonine. It was reported to have similar function to serine-threonine dehydratase, a versatile catalyst that functions as a coenzyme in a multitude of reactions, including amino–sugar breakdown [50]. Others were: sucrose synthase, formate dehydrogenase, biotin, and lipoic acid-binding proteins, which may serve as sources of energy for the pathogen. Additional up-regulated proteins were serine-threonine kinases, possibly *P. infestans* secreted kinases [51]. Calcium-dependent protein kinase, arginine N-methyltransferase (their function in the late stage disease process is unknown) and Tyrosine Phosphatase (reported in bacteria is an effector protein), when overexpressed, significantly increases bacterium *Pseudomonas syringae* virulence [52,53]. 

Several studies have reported that genes encoding hydrolytic enzymes, such as serine protease, glucosidases, glucanases, and lyases, constituted a major portion of Phytophthora potential pathogenicity factors [48]. Among the significantly induced pathogenesis factors were enzymes: beta-glucanase, glycosyl hydrolases, cysteine protease, and pectate lyase. We also noticed ATP synthase and proton ATPase transmembrane transporter; their specific role in pathogenesis is unknown.

Notable significantly down-regulated proteins were those involved in the structural integrity of the ribosome, such as ribosome recycling factor domain proteins, 60S ribosomal protein L18a, acidic ribosomal protein P1a-like, 50S ribosomal protein L32, and ribosomal-L12 proteins. We noticed other proteins with catalytic and binding domain functions, such as cellulose synthase, histone H2A, Nicotinamide adenine dinucleotide phosphate (NADPH)-protochlorophyllide oxidoreductase, pectinesterase, endoglucanase, FK506-binding protein, and PPM-type phosphatase domain. The latter being dephosphorylate serine and threonine residues. Among the down-regulated proteins were also: thioredoxin-like protein CITRX a chloroplastic protein, demonstrated to be involved in the negative regulation of cell death and tomato Cf-9 resistance protein function by specifically interacting with Cf-9 [54]. Silencing of CITRX accelerated the Cf-9/Avr9-triggered hypersensitive response in both tomato and *Nicotiana benthamiana*, together with the enhanced high accumulation of reactive oxygen species, and the induction of down-stream defense-related genes. In the same study, silencing of CITRX also conferred increased resistance to the fungal pathogen *Cladosporium fulvum* in susceptible Cf0 tomato [54,55]. Several uncharacterized proteins with acetyltransferase-like domain proteins, including M1BC65, a member of the chloramphenicol acetyltransferase-like domain superfamily, was also identified. In addition to enzyme inhibitor and peptide regulator proteins, such as Clone PI9149 apoptosis inhibitor 5-like protein and Proteinase inhibitor I, the high number of down-regulated DEPs (443/723) among the proteins in the LI/EI seems consistent with a shut-down of the cellular metabolic process caused by HR induced necrosis. Collectively, the cellular metabolic process, protein folding, and modification processes, including cell wall re-organization, seem to be the most common roles of the LI/EI-DEPs; therefore, we speculate that these proteins play an essential role in the biological processes possibly involved in disease conditioning and the shut-down of cell metabolic processes at the late infection stage, which are geared towards containment of the invading pathogen.

#### 2.4.3. Differential Regulation of Commonly Shared Proteins in Response to *P. infestans* during Early and Late Disease Stages

Our comparative analysis identified 18 proteins shared between the EI/Control comparison and LI/EI comparison (Figure 2b, Table S7). We reasoned that these common set of proteins might respond to the same signal that controls the switch from general plant defense induction, based on PAMPs (PTI), to effector-triggered immunity (ETI), and might have a similar pattern of expression. Indeed, 10 out of the 18 common DEPs showed a similar expression profile throughout the time course. For instance, they were up-regulated explicitly from Control to EI and reached their maximum expression level by time-point LI. Based on GO analysis, these 10 proteins were mostly located within the extracellular region, cell membrane, and protein-containing complexes, and are involved in energy production, vesicle-mediated transport, and response to oxidative stress. From this group we noticed two uncharacterized proteins, M0ZTQ4 (FC = 2.40) and M1CUM0 (FC = 5.6), with at least two- and five-fold increase in abundance. The M0ZTQ4 contained the osmotin/thaumatin-like domain, and osmotins are members of pathogenesis-related proteins, secreted into the cell wall to promote basal resistance responses [56]. Whereas M1CUM0 has a domain function related to terpene biosynthesis, a part of antifungal phytoalexins shown to limit the growth hypha during pathogen invasion [57,58].

Remarkably, six out of the remaining eight DEPs (M1ABL9, M1D1L9, M1A3A0, M1A4R1, M1C8Q0, M0ZZ55) showed a dynamic reprogramming in response to *P. infestans*. It is noteworthy that after inoculation these proteins were up-regulated from timepoint Control to EI and reached their highest expression level at time-point EI, afterward they were significantly repressed (Table S7). Further analysis showed that they contained vacuolar protein sorting-associated VPS4 binding domain, ribosomal protein L24 binding domain, pentatricopeptide repeats, histone H1/H5 globular binding domain, 26S proteasome subunit RPN7 domain, and vesicle transport protein, respectively. Generally, plants activate numerous defense mechanisms that can contribute to resistance against pathogen invasion. Considering that a significant increase in lesions’ size was observed on the leaves of cv. Sarpo Mira at the LI time-point, compared to the EI timepoint, it follows that the dynamic reprogramming of these proteins might be very significant. This highlights the tight regulation of defense activities within the host cell and demonstrates the urgency for cell wall modification and reinforcement, as well as an efficient transport mechanism for defense-related compounds upon pathogen invasion consistent with the incompatible pathogen–host interaction and observed phenotype in Figure 1a,b.

Contrastingly, two DEPs, K7WNX1 and M1C639, were consecutively down-regulated throughout the whole-time course; K7WNX1 is a light-inducible tissue-specific ST-LS1 protein and M1C639 contained the transmembrane helix domain. Their role in potato–*P. infestans* interaction is unknown.

### 2.5. Validation of Differentially-Expressed Proteins in Early and Late Disease Stages

**Figure 6:**
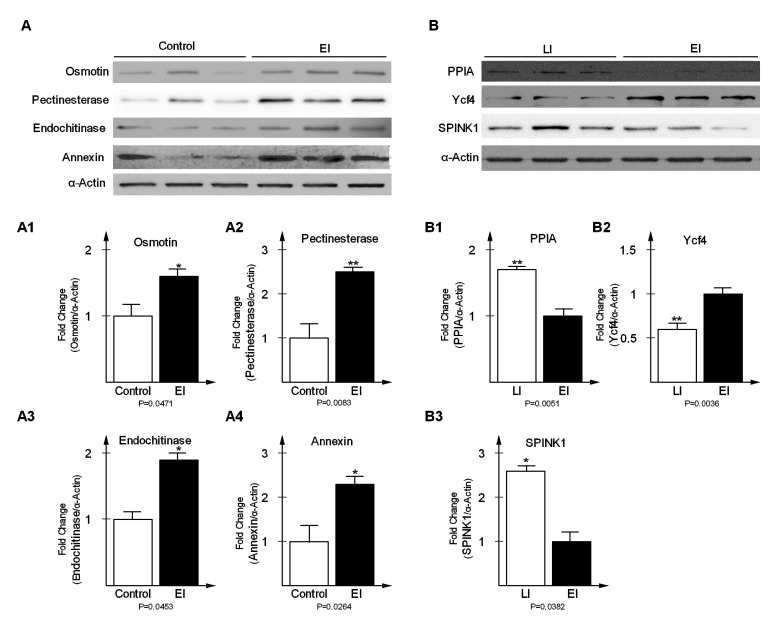
Western blot analysis of differentially expressed proteins at early disease stage (**A**) and late disease stage (**B**). Expression levels of Osmotin (fold change = 1.62, *p* = 0.00471), Pectinesterase (fold change = 2.53, *p* = 0.0083), Endochitinase (fold change = 1.94, *p* = 0.0453) and Annexin (fold change = 2.37, *p* = 0.0264) were significantly increased in EI relative to Control (*A1*, *A2*, *A3*, *A4*); PPIA (fold change = 1.74, *p* = 0.0051) and SPINK1 (fold change = 2.63, *p* = 0.0382) were significantly increased in LI relative to EI (*B1*, *B3*); Ycf4 (fold change = −1.67, *p* = 0.0036) was significantly decreased in LI relative to EI and (*B2*). Potato actin represented loading control.

To validate DEPs from the time series proteomics experiments, a total of seven proteins were selected, of which four were randomly selected from the early disease stage and the remaining three were selected from late stage, to verify the expression level, via western blot analysis (Figure 6). Osmotin (fold change = 1.62, *p* = 0.00471), pectinesterase (fold change = 2.53, *p* = 0.0083), endochitinase (fold change = 1.94, *p* = 0.0453), and annexin (fold change = 2.37, *p* = 0.0264) were significantly up-regulated in EI relative to the Control (Figure 6(A1)–(A4)). In the late stage, peptidyl-prolyl cis-trans isomerase (fold change = 1.74, *p* = 0.0051) and type I serine protease inhibitor (fold change = 2.63, *p* = 0.0382) were significantly increased in LI relative to EI (B1, B3). In contrast, photosystem I assembly protein Ycf4 (fold change = −1.67, *p* = 0.0036) was significantly down-regulated relative to EI (Figure 6(B2)). Potato actin represented loading control. The western blot results were consistent with the times series proteomics data, which strongly support the reliability of the results reported in this paper.

### 2.6. Essential Proteins for Early and Late Disease Stages

In this study, we systematically identified several proteins that are important for the potato defense response against *P. infestans* at the early and late stages of infection, including those which may otherwise contribute to pathogenesis. The criteria described in the Methods allowed us to select several proteins that play a significant role in both disease stages, which are listed in Table 1 and some of which are described hereafter.

#### 2.6.1. Cv. Sarpo Mira Protein–Protein Interaction during the Early Stages of Infection

**Figure 7:**
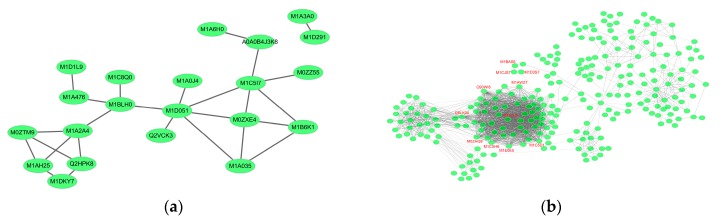
Network analysis results for significantly changed proteins in the (**a**) early and (**b**) late stages of infection.

Most proteins carry out essential biological functions, like signal transduction, protein modification, cellular metabolism, cytokinesis, DNA replication, RNA transcription, and targeted degradation, by interacting with other proteins in a protein complex. To uncover functional interactions among proteins during the early stages of *P. infestans* invasion, we analyzed the 75 DEPs in the EI/Control comparison by Search Tool for the Retrieval of Interacting Genes/Proteins (https://string-db.org) in Cytoscape (Figure 7a). Of interest, was the association among nine proteins: wall-associated kinase protein (WAKP, M1D051), an integral component of the cell membrane; a ubiquitin-conjugating enzyme protein (M1BLH0); a chloroplast nucleoid DNA-binding protein (M1B6K1); a ribosomal L24 domain (M1D1L9); a 26S proteasome subunit-containing RPN7 protein domain (M1C8Q0); endochitinase (Q2HPK8); osmotin (M0ZTM9); a pathogenesis-related PR1 protein containing a RlpA-like double-psi beta-barrel domain (M1A2A4); and finally, protein disulfide isomerase inhibitor (PDI, M1C517).

We reasoned that *P. infestans* might directly or indirectly elicit the induction of these proteins, and their association was important to promote coordination of several processes, including signal transduction, cellular metabolic process, and defense against *P. infestans*. For example, WAKP (M1D051, FC = 1.28) was predicted as a major functional node having multiple protein interactions, and it was specifically significantly up-regulated from Control to EI time-points, through to LI. Previous studies have shown that WAK proteins are important oligogalacturonide receptors, required for the activation of the plant immune response, as well as for growth and development [59]. Additionally, there are reports of the arabidopsis RFO1 gene coding for a wall-associated kinase protein that conferred quantitative resistance against *Fusarium oxysporum* [60]. In the present study, it is likely that WAKP, in association and coordination with other proteins, acted as an elicitor by binding to damage-associated molecular patterns (DAMPs) or cell wall defense proteins [61,62]. We also noticed that a putative ribosomal protein in the network was highly abundant (M1D1L9, FC = 1.36), and a protein possessing ubiquitin-binding domain (M1C8Q0, FC = 1.22). Previous studies have reported the involvement of these two proteins in *P. parasitica*–tomato interaction [63]. Next are the constitutively highly-expressed Endochitinase proteins (Q2HPK8, FC = 1.43; M1AH25, FC = 1.33), which have common function in defense-related signaling to boost the non-specific defense response by releasing elicitor-active chitin oligomers [60]. It has been shown that basic chitinase and osmotin-like protein possess actin-binding capabilities and cooperate to promote cytoplasmic aggregation in the potato cells, as a defense against penetration of Phytophthora [64]. In the present study, the significant up-regulation of these several osmotins, (M0ZTM9, FC = 1.34) from Control to EI, and through to LI, suggest significant coordination among pathogenesis-related proteins within the cell wall to promote basal resistance responses. Indeed, the PR1 gene was implicated in basal resistance against *P. infestans* oomycete [65]. In this study, PR1 protein (FC = 1.30) was up-regulated, its expression level was highest at the late stage (1.40), which indicates that this protein was significantly induced throughout the time course, which is consistent with previous studies [66]. Also, the KiTH-2 protein was strongly up-regulated (FC =1.82) upon infection with *P. infestans*; its specific role in potato–*P. infestans* interaction or pathogen resistance is not clear [67]. Although it belongs to the Kiwellin family and contains the rare lipoprotein A (RlpA) domain, which has been shown to act as a prc mutant suppressor in *Escherichia coli* [68]. Here, we speculate that it may function as part of elicitor machinery during pathogen infection.

Meanwhile, studies have shown that the PDI gene (FC = 1.31, M1C517) plays a crucial role in host–pathogen interaction and that PDI protein is localized to the haustoria of Phytophthora, a major site of pathogen protein export into the host cell during infection [69]. In the present study, the significant up-regulation of PDI from Control to EI, and subsequent decrease in abundance at LI, suggests this protein might be acting as a virulence factor of *P. infestans* at the early infection stage, and potentially contributes to plant infection and the late-stage disease process [70]. Additionally, the plant’s secretory system is crucial for building resistance at the cell periphery. It also enables attacked cells to transport antimicrobial compounds and cell wall material to the site of attempted penetration [38]. In this study, we found a vesicle-mediated transport protein (M0ZZ55, FC = 1.77) containing the v- SNARE domain, significantly up-regulated from Control to EI, and with stable expression afterward, which suggests a tightly coordinated transport process during early stages of pathogen attack, which has been previously reported [69]. Together, these results show that during the early stages of disease infection there was a coordinated up-regulation of defense arsenal, like the pathogen recognition proteins, signaling molecules, antimicrobial compounds, cellular trafficking, primed to initiate a broad-based resistance against *P. infestans*.

#### 2.6.2. Cv. Sarpo Mira Protein–Protein Interaction during the Late Stages of Infection

We analyzed the protein interaction of DEPs identified in LI/EI comparison and found multifactorial interactions with several hub proteins in the network (Figure 7b). Prominent among the proteins in the network was urease (Q93WI8, FC = 1.27), with over 67 connections. In this study, urease increased slightly in abundance from Control (avg. = 0.86) to EI (avg. = 0.90), but was significantly induced at LI (avg. = 1.14). Studies have shown that urease inhibits the growth of phytopathogenic fungi [71,72]. We also found an uncharacterized protein, M1E0E5 (FC = 1.22), with 57 connections, which contained a pyridoxal-phosphate-binding site, and was predicted to participate in aminotransferase activity, oxidoreductase, and mononucleotide binding. Similarly, we observed two other proteins (57 connections) coding for Hydroxyacyl-CoA dehydrogenase (M0ZHQ8, FC = 1.26), with functions related to oxidoreductase activity. Other proteins included metalloenzyme (39 connections, O81394, FC = −1.20), involved in catalytic conversion of 2-phosphoglycerate to phosphoenolpyruvate; GA3PDH enzyme (34 connections, Q8LK04, FC = 1.20), involved in glycolysis and gluconeogenesis; citrate synthase protein (32 connections, M1AD15, FC = 1.38); histidine kinase/HSP90-like ATPase (32 connections, M1C5D1, FC = 1.21); thioredoxin (28 connections, M1CXH6, FC = 1.24); and glutathione peroxidase (20 connections, M1AWZ7, FC = 1.37).

The late stage of the *P. infestans* disease process is usually characterized by colonization and necrotrophy [48]. At this stage, it is also likely that the pathogen faced a shortage of nutrients, like glucose, fatty acid, amino acids, and energy, from the shut-down of the host’s cellular metabolism by HR-induced cell death, and may attempt to induce host metabolic changes to enable nutrient supply and growth. For example, GA3PDH was up-regulated and is required for the breakdown of glucose for energy and carbon molecules. Hydroxyacyl-CoA dehydrogenase is essential for fatty acid beta-oxidation, which can also provide energy. While pyridoxal phosphate P5P is an active form of vitamin B6, which is known to be involved in various reactions, including amino acid break-down, transamination, decarboxylation, and racemization reactions. It is likely that P5P acts to counteract the toxic effects of the oxidative burst and, alternatively, to break down amino acids for energy, possibly contributing to pathogenesis. Additionally, for the pathogen colonization to succeed, it must inactivate or remove the host-induced reactive oxygen species (ROIs), and thioredoxin proteins can counteract or protection the pathogen against ROIs [48]. Other proteins that showed sparse interaction included: cytochrome P450 proteins, possibly involved in efflux and detoxification [62]; and anamorsin; interestingly, anamorsin has been reported to be involved in negative regulation of apoptosis as well as cellular iron homeostasis [73]. Likewise, we noticed glycosyl hydrolase, a cell wall-degrading enzyme, was probably involved in virulence as well as nutrition or growth at the late disease stage.

We also identified glutathione S-transferase and glutathione peroxidase, which were previously reported to be involved in pathogen antioxidant defenses and host cell detoxication [62,74]. Based on these results we hypothesize that in an incompatible interaction some of the DEPs at the late stage may have a function-related late-stage disease-susceptible process. Notwithstanding, a strong domain self-interaction was observed among the cell death regulator proteins (M1D3S7, M1CJS7, M1BAS6) at this stage.

#### 2.6.3. Correlation Analysis of Protein Expression and mRNA by Real Time-qPCR

**Figure 8:**
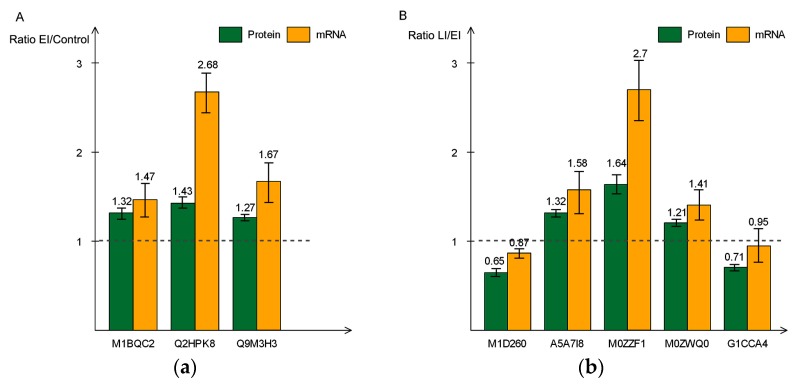
Real-time polymerase chain reaction (PCR) quantitative analysis of eight differentially-expressed proteins and mRNA at early (**a**) and late (**b**) disease stages. The green bar indicates the expression level determined by Tandem mass tag (TMT) and orange bar shows relate expression of mRNA. All data are presented as mean ± SD (*n* = 3 in each time-point).

In order to evaluate the correlation between mRNA and protein levels, we analyzed the relative expression pattern of genes encoding eight representative proteins in the context of the three time-points by RT-qPCR. For each of the eight genes, we found that the transcript level increased in the early and late stage of the disease process, in agreement with their protein expression levels, as revealed by TMT data. For example, in the early stages of disease infection, three of the eight genes, coding for proteins involved in cell wall structure modification, had a higher expression level (Figure 8a). A similar pattern was observed for the remaining five genes at the later stage of the infection cycle (Figure 8b). These results validate the increased levels of the encoded proteins that were observed in the proteomic analysis and suggest that the abundance of these proteins is likely regulated at the transcriptional level.

In summary, this study sheds light on the changes in protein abundance and physiological roles of proteins in potato during the early and late stages of *P. infestans* oomycete infection. We have also inferred protein interaction that occurred during the two disease stages, either physical interactions verified through experiments, or predictions to further understand the disease process. Overall, our analysis suggests that differentially-expressed proteins, identified at the early stage of infection, played significant roles in signal transduction and the basal defense response, while the late stage disease process was characterized by the significant abundance of R proteins related to disease resistance processes and cell death. However, some of proteins in the late disease stage could be related to late-stage disease-susceptible processes. Therefore, the data reported here is a valuable resource for practical use to further characterize the mechanisms that are potentially involved in the potato late blight disease resistance process.

## 3. Materials and Methods

### 3.1. Plant Material and Growth Conditions

Potato plants of cultivar Sarpo Mira were grown in a greenhouse with controlled conditions set at 20 °C, 16:8 light to dark cycle, and 70% relative humidity. Five-week-old plants were transferred to an infection chamber with 100% humidity and 10:14 light:dark cycle. After 6 h, plants were sprayed with an encysted zoospore suspension from *P. infestans* isolate until the leaf surfaces were fully saturated with the zoospore suspension (15,000 sporangia/mL). Samples were collected at 0, 48, and 120 h post inoculation (hpi) according to [75], and labelled as Control, EI, and LI, respectively. The 0 dpi samples were collected immediately after inoculation with a contact time of less than one minute [75]. Afterwards, the relative humidity was maintained at 100% for two days after inoculation and then adjusted to 90% for the rest of the experiment. For each time-point, samples of fully-expanded upper leaves were collected from three independent biological experiments. All the materials were frozen in liquid nitrogen and stored at −80 °C until use [31].

### 3.2. Protein Extraction

For each sample, 1 g was weighed and homogenized by grinding in liquid nitrogen and transferred to a 50 mL precooled test-tube. Afterwards 25 mL of precooled acetone (−20 °C), containing 10% (*v*/*v*) trichloroacetic acid (TCA) and 65 mM dithiothreitol (DTT), was added. After thorough mixing, the homogenate was precipitated for 2 h at −20 °C and then centrifuged for 30 min at 16,000× *g* at 4 °C. The supernatant was carefully removed, and the pellet was rinsed three times with 20 mL of cold acetone (−20 °C), followed by centrifugation (20,000× *g* for 30 min at 4 °C). The precipitation was collected and vacuum freeze-dried. A 250 mg sample of the freeze-dried pellets was weighed and placed in a 1.5 mL Eppendorf tube. The pellets were dissolved in SDT lysis buffer (4% SDS, 100 mM Tris-HCl, 100 mM DTT, pH 8.0) and then boiled for 5 min. After boiling and vortex mixing for 30 s, the mixture was intermittently sonicated in an ice bath, with 5 s sonication followed by 10 s break, for 5 min at 100 W. The mixture was then boiled again for 5 min, followed by 30 min centrifugation (12,000× *g*, 20 °C). The supernatant was collected in a new 1.5 mL Eppendorf tube, filtered through a 0.22-μm Millipore filter and collected as lysate. Protein concentration in the lysate was determined using the bicinchoninic acid (BCA) protein assay reagent (Beyotime Institute of Biotechnology, Shanghai, China). The rest of the lysate was frozen at −80 °C until use.

### 3.3. Protein Digestion

TMT analysis was performed according to the method described by [76], Briefly, protein concentrates (300 µg) in an ultrafiltration filtrate tube (30 kDa cut-off, Sartorius, Gottingen, Germany) was mixed with 200 µL Urea buffer (8 M urea, 150 mM Tris-HCl, pH 8.0) and the sample was centrifuged at 14,000× *g* at 20 °C for 30min. The sample was washed twice by adding 200 µL UA and centrifuged at 14,000× *g* at 20 °C for 30min. The flow-through from the collection tube was discarded. Next, 100 µL Indole-3-acetic acid (IAA) solution (50 mM IAA in UA buffer) was added to the filter tube and vortexed at 600 rpm in a thermomixer comfort incubator (Eppendorf, Germany) for 1 min. Subsequently, the sample was incubated at room temperature for 30 min in the dark and spun at 14,000 g for 30 min at 20 °C. Next 100 µL UA was added to the filter unit and centrifuged at 14,000 g for 20 min, and this step was repeated twice. The protein suspension in the filtrate tube was subjected to enzyme digestion with 40 µL of trypsin (Promega, Madison, WI, USA) buffer (4 µg trypsin in 40 µL of dissolution buffer) for 16–18 h at 37 °C. Finally, the filter unit was transferred to a new tube and spun at 14,000 g for 30 min. Peptides were collected in the filtrate and concentration of the peptides was measured by optical density with a wavelength of 280 nm (OD280).

### 3.4. TMT Labeling and LC-MS/MS Analysis

Approximately 50ug of digested peptides from each sample, including the internal standard, were labeled with TMT reagents (Thermo Fisher Scientific, San Jose, CA, USA) following procedures recommended by the manufacturer. Briefly, peptides from the samples LI1, LI2, LI3, Control 1, Control 2, Control 3, EI 1, EI 2, and EI3 were labeled with TMT reagents 126, 127N, 127C, 128N, 128C, 129N, 130N, 130C, and 131, respectively. All labeled peptides were pooled together. Labeled and mixed peptides were subjected to high-pH reversed-phase fractionation in the 1100 Series High-performance liquid chromatography Value System (Agilent, Palo Alto, CA, USA) equipped with a Gemini-NX (Phenomemex, 00F-4453-E0) column (4.6 × 150 mm, 3 µm, 110 Å). Peptides were eluted at a flow rate of 0.8 mL/min. Buffer A consisted of 10 mM ammonium acetate (pH10.0) and buffer B consisted of 10 mM ammonium acetate and 90% *v*/*v* Acetonitrile (pH 10.0). Buffer A and B were both filter-sterilized. The following gradient was applied to perform separation: 100% buffer A for 40 min, 0%–5% buffer B for 3 min, 5%–35% buffer B for 30 min, and 35%–70% buffer B for 10 min. Then, 70%–75% buffer B for 10 min, 75%–100% buffer B for 7 min, 100% buffer B for 15 min, and 100% buffer A for 15 min. The elution process was monitored by measuring absorbance at 214 nm, and fractions were collected every 75 s. Finally, the collected fractions (approximately 40) were combined into 10 pools. Each fraction was concentrated via vacuum centrifugation and was reconstituted in 40 µL of 0.1% *v*/*v* trifluoroacetic acid. All samples were stored at −80 °C until further analysis.

The TMT-labeled samples were analyzed using easy-nLC nanoflow HPLC system connected to an Orbitrap Elite mass spectrometer (Thermo Fisher Scientific, San Jose, CA, USA). A total of 1 µg of each peptide sample was loaded onto Thermo Scientific EASY column (two columns) using an autosampler at a flow rate of 150 nL/min. The sequential separation of peptides on Thermo Scientific EASY trap column (100 µm × 2 cm, 5 µm, 100Å, C18) and analytical column (75 μm × 25 cm, 5 μm, 100 Å, C18) was accomplished using a segmented 2 h gradient from Solvent A (0.1% formic acid in water) to 35% Solvent B (0.1% formic acid in 100% Acetonitrile) for 100 min. Followed by 35%–90% Solvent B for 12 min and then 90% Solvent B for 8 min. The mass spectrometer was operated in positive ion mode, and MS spectra were acquired over a range of 350–2000 *m*/*z*. Resolving powers of the MS scan and MS/MS at 100 *m*/*z* for the Orbitrap Elite were set as 60,000 and 15,000, respectively. The top sixteen most intense signals in the acquired MS spectra were selected for further MS/MS analysis. The isolation window was 1 *m*/*z*, and ions were fragments through higher energy collisional dissociation with normalized collision energies of 35 eV. The maximum ion injection time was set at 50 ms for the survey scan, and 150 ms for the MS/MS scans, and the automatic gain control target values for full san modes was set to 10 × 10^−6^, and for MS/MS it was 5 × 10^4^. The dynamic exclusion duration was 30 s.

### 3.5. Database Search, Protein Identification, and Quantification

The Proteome Discoverer 2.1 (Thermo Fisher Scientific) was used to analyze raw data. The Mascot 2.1 (Matrix Science) embedded in Proteome Discoverer was used to search raw data against the UniProt potato database (December 21, 2017; 55,715 sequences). Search parameters were as follows: monoisotopic mass; trypsin as cleavage enzyme; two max missed cleavages; TMT 10plex (N-term), TMT 10plex (K), and carbamidomethylation of cysteine as fixed modifications; and oxidation of methionine as variable modifications. Peptide mass tolerance of ±20 ppm and fragment mass tolerance of 0.1 Da were used for parent and monoisotopic fragment ions, respectively. Results were filtered based on a false discovery rate of (FDR) ≤0.01. Relative quantitative analyses of proteins were based on ratios of TMT reporter ions from all unique peptides representing each protein. For protein quantitation, each reporter ion channel was summed across all quantified proteins and normalized assuming equal protein loading of all ten samples. The protein ratios of each sample were normalized to the TMT-126 label [77]. The mass spectrometry proteomics data are available at ProteomeXchange Consortium via the PRIDE [78] partner repository with identifier PXD010045.

### 3.6. Bioinformatics and Statistical Analysis

Proteome Discoverer 2.1 Protein quantitation values were exported for further analysis in Excel. Proteins of *p*-values <0.05 by Student *t*-test and a fold-change of >1.20 or <0.83 in expression between any two groups were considered significant. Differentially expressed proteins (DEPs) were classified by their gene functions and also by biological pathways using the publicly available gene ontology (GO) database provided by the Gene Ontology Consortium (http://geneontology.org/) [79]. The identified protein sequence information was extracted from the UniProt knowledge base and retrieved in FASTA format. The functional information of the homologous proteins was used to annotate targeted proteins. Top 10 blast hits with E-values of less than 1e-3, for each of the query proteins, were retrieved and loaded into Blast2GO (Version 2.7.2) [80], a high-throughput online tool for gene ontology (GO) analysis, for GO mapping, and annotation. Enriched GO terms were identified with Fisher’s exact test and hypergeometric distribution test cutoff of 0.05. Information on the biological pathways was obtained from the Kyoto Encyclopedia of Genes and Genomes pathways database (http://www.genome.jp/kegg/pathway.html) [81]. Visualization of these pathways and enrichment analysis was performed using the KOBAS 2.0 software [82,83,84]. *p* < 0.05 was set as the threshold used for enrichment analysis of KEGG pathways. Interactions among differentially-expressed proteins in early and late disease stages were analyzed by Cytoscape software, and were used to draw the protein interaction network [82].

### 3.7. Antibodies and Western Blot Analysis

For western blot analysis, the procedures of electrophoresis, transfer, and immunodetection were performed according to Howden et al. [85]. The primary antibodies used were as follows: antibody for the Osmotin-like protein (Q5XUH0, Biorbyt orb27915, 1:1000); pectinesterase (M1BQC2, PLLABS PL0304687, 1:500); endochitinase (Q2HPK8, PhytoAB PHY1514S, 1:2000); annexin (Q9M3H3, PhytoAB PHY0729S, 1:2000); peptidyl-prolyl cis-trans isomerase (M0ZZF1, PhytoAB PHY0920S, 1:2000); photosystem I assembly protein Ycf4 (G1CCA4, PhytoAB PHY1363S, 1:1500); type I serine protease inhibitor (E0WCF2, PhytoAB PHY0146S, 1:2000). Horseradish peroxidase-conjugated anti-rabbit IgG (dilution 1:15,000, Bio-Rad, Hercules, CA, USA) were used as secondary antibodies. After immunodetection, the intensity of the immuno-stained bands were normalized for the total protein intensities measured by Coomassie blue from the same [86]. The images were subjected to densitometric analysis performed using Quantity One software (Bio-Rad).

### 3.8. Criteria for Selecting Essential Proteins

Candidate proteins were selected belonging to the following groups: (a) Common and unique DEPs belonging to the early disease stage, chosen based on their protein abundance, fold change value, and functional category enrichment. Additionally, candidates were further selected based on the inferred protein interaction as well as those with putative function that are relevant to late blight disease infection response during early infection. Furthermore, previously reported proteins were also considered in candidate gene selection in both stages. (b) Common and unique DEPs from the late disease stage, chosen based on expression profile, fold change value, functional category enrichment, and putative function relevant to the late disease stage disease process, in addition to the inferred protein interactions in the network.

### 3.9. Correlation between mRNA and Protein Levels by Quantitative Real-Time Polymerase Chain Reaction (qRT-PCR)

Leaf samples were collected at 4, 48, and 120 h for protein and total RNA isolation. Total RNA was extracted from each sample using TRIzol Reagent Kit (AmbionTM) according to the manufacturer’s instructions. Next, each of the RNA samples was treated with RNase-free DNase (Takara, Dalian, China). Complementary DNA (cDNA) was retro-transcribed from 2 μg of total RNA using the Thermo Scientific RevertAid Kit according to the manufacturer’s instructions. Quantitative real-time polymerase chain reaction (qPCR) was performed on a Bio-Rad real-time detection system (Bio-Rad). PCR conditions were 95 °C for 1 min, followed by 44 cycles at 95 °C, 12 s, 60 °C, 30 s, and 72 °C, 30 s. After cycling, melting curves of the reaction were run from 55 °C to 95 °C. Each reaction was performed in three technical replicates, and the expression profiles of 8 genes were analyzed, with potato ef1a gene used as the constitutive gene for normalization. The quantification of gene expression levels was calculated relative to ef1a with the 2^−ΔΔ*C*T^ method [87]. Primer sets used for qRT-PCR are reported in Table S8.

## Figures and Tables

**Table 1:** Proposed candidate proteins that played an essential role at the early and late disease stages.

Accession	Description	Fold Change	*p* Value	GO Names
**Early stage up-regulated proteins**				
M0ZZ55	Uncharacterized protein containing longin N-terminal domains with C-terminal coiled-coil/SNARE motif.	1.77	0.02	C: integral component of membrane; P: vesicle-mediated transport
M0ZTM7	Osmotin	1.58	0.03	
Q5XUH0	Osmotin-like protein	1.54	0.002	
M0ZMK7	Uncharacterized protein with domain named as Bet v 1. Bet v 1 belongs to family 10 of plant pathogenesis-related proteins (PR-10)	1.51	0.004	P: defense response; P: response to biotic stimulus
P52402	Glucan endo-1,3-beta-glucosidase, basic isoform 3 (Fragment)	1.45	0.03	F: hydrolase activity, hydrolyzing O-glycosyl compounds; P: carbohydrate metabolic process
Q2HPK8	Putative endochitinase (Fragment)	1.43	0.04	F: chitinase activity; P: chitin catabolic process; P: cell wall macromolecule catabolic process
M1D1L9	Uncharacterized protein containing domain 2 of Ribosomal protein L2,	1.36	0.02	F: structural constituent of ribosome; C: ribosome; P: translation
M1CUM0	Uncharacterized protein containing a domain found in the isoprenoid synthase family	1.34	0.04	F: magnesium ion binding; P: metabolic process; F: terpene synthase activity
M1D578	Peroxidase	1.34	0.04	F: peroxidase activity; P: response to oxidative stress; F: heme binding; P: hydrogen peroxide catabolic process; P: oxidation-reduction process
M1BQC2	Pectinesterase	1.32	0.03	F: enzyme inhibitor activity; C: cell wall; F: pectinesterase activity; P: cell wall modification
M1A3A0	Uncharacterized protein containing Pentatricopeptide repeat (PPR)	1.29	0.02	F: protein binding
M1A035	Carboxypeptidase	1.28	0.03	F: serine-type carboxypeptidase activity; P: proteolysis
M1D051	Wall-associated kinase	1.28	0.03	F: protein kinase activity; F: ATP binding; P: protein phosphorylation
Q9M3H3	Annexin	1.27	0.01	F: calcium ion binding; F: calcium-dependent phospholipid binding
M0ZXE4	Uncharacterized protein contains a domain found in serine peptidases	1.27	0.02	F: serine-type endopeptidase activity; P: proteolysis
Q4FE26	Proteinase inhibitor 1 PPI3B2	1.26	0.03	F: serine-type endopeptidase inhibitor activity; P: response to wounding
M1ABL9	Uncharacterized protein contains AAA+ ATPase domain	1.25	0.003	F: ATP binding and hydrolysis
Q84XQ4	NtPRp27-like protein	1.25	0.02	
M1A4R1	Uncharacterized protein with AT hooks a DNA-binding motif	1.24	0.004	C: nucleosome; F: DNA binding; C: nucleus; P: nucleosome assembly
M1C8Q0	Uncharacterized protein contains Proteasome component Initiation (PCI) domain	1.22	0.02	F: enzyme regulator activity
**Early stage down-regulated proteins**				
K7WNX1	Light-inducible tissue-specific ST-LS1	0.83	0.001	C: photosystem II oxygen-evolving complex; P: photosynthesis
M0ZJ30	Uncharacterized protein contains Major facilitator domain	0.74	0.002	C: membrane; F: transmembrane transporter activity; P: transmembrane transport
M1BNJ1	Uncharacterized protein contains Carotenoid oxygenase binding site.	0.68	0.05	F: oxidoreductase activity, acting on single donors with incorporation of molecular oxygen, incorporation of two atoms of oxygen; P: oxidation-reduction process
**Late stage up-regulated proteins**				
M1BD67	Uncharacterized protein with Acid phosphatase, class B-like domain	5.68	0.03	F: acid phosphatase activity
M0ZTQ4	Uncharacterized protein predicted with Osmotin/thaumatin-like domain	5.62	0.05	
M1CJS7	Uncharacterized protein contains CC-NB-ARC and LRR Domains.	4.00	0.01	F: ADP binding
M1B864	Uncharacterized protein contains Kunitz inhibitor STI-like domain.	3.65	0.04	F: endopeptidase inhibitor activity
M1AU65	Peroxidase	2.94	0.01	F: peroxidase activity; P: response to oxidative stress; F: heme binding; P: hydrogen peroxide catabolic process; P: oxidation-reduction process
E0WCF2	PIN-A	2.92	0.0001	F: serine-type endopeptidase inhibitor activity; P: response to wounding
A0A097H185	PIN-I-Protein	2.52	0.01	F: serine-type endopeptidase inhibitor activity; P: response to wounding
M0ZZF1	Peptidyl-prolyl cis-trans isomerase	1.64	0.02	P: protein peptidyl-prolyl isomerization; F: peptidyl-prolyl cis-trans isomerase activity
M1BAS6	Uncharacterized protein (CC-NB-ARC and LRR Domains)	1.62	0.03	F: ADP binding
Q8H9B9	Hyoscyamine 6-beta-hydroxylase-like protein (Fragment)	1.62	0.03	F: oxidoreductase activity; P: oxidation-reduction process
A5A7I8	Calcium-dependent protein kinase 5	1.32	0.04	F: protein kinase activity; F: calcium ion binding; F: ATP binding; P: protein phosphorylation
M1AKD9	Uncharacterized protein contains a Histidine kinase/HSP90-like ATPase domain	1.30	0.02	F: ATP binding; P: protein folding; P: response to stress; F: unfolded protein binding
Q8VX50	Putative receptor-like serine-threonine protein kinase	1.22	0.05	F: protein kinase activity; F: ATP binding; P: protein phosphorylation
M1D3S7	Uncharacterized protein contains a CC-NB-ARC and LRR Domain.	1.22	0.05	F: ADP binding
M0ZWQ0	V-type proton ATPase proteolipid subunit	1.21	0.03	F: proton transmembrane transporter activity; P: ATP hydrolysis coupled proton transport; C: proton-transporting V-type ATPase, V0 domain
**Late stage down-regulated proteins**				
K7WNV9	Thioredoxin	0.82	0.05	P: glycerol ether metabolic process; F: protein disulfide oxidoreductase activity; P: cell redox homeostasis
G1CCA4	Photosystem I assembly protein Ycf4	0.71	0.03	C: photosystem I; P: photosynthesis; C: integral component of membrane
M1D260	FK506-binding protein	0.63	0.03	P: histone peptidyl-prolyl isomerization; F: peptidyl-prolyl cis-trans isomerase activity; F:FK506 binding; C: nucleolus
M1B8N6	NADPH-protochlorophyllide oxidoreductase	0.37	0.04	F: protochlorophyllide reductase activity; P: oxidation-reduction process

## Supplementary Materials

Supplementary materials can be found at http://www.mdpi.com/1422-0067/20/1/136/s1. Table S1: List of all proteins identified in the time series analysis. Table S2: List of all differentially expressed proteins, functional annotation, significantly enriched INTERPRO protein domains and features, PFAM domains, and KEGG pathways classification. Table S3: List of differentially-expressed proteins during the early stage of *P. infestans* infection. (EI/Control). Table S4: List of differentially-expressed proteins during the late stage of *P. infestans* infection. (LI/EI). Table S5: List of differentially-expressed proteins from time-point LI/Control. Table S6: Gene Ontology classification of differentially-expressed proteins. Table S7: List of common differentially-expressed proteins throughout the whole time course of the experiment. Table S8: List of primer sets used in the qRT-PCR correlation analysis of protein expression and mRNA.
